# Consumption of Dehulled Adlay Improved Lipid Metabolism and Inflammation in Overweight and Obese Individuals after a 6-Week Single-Arm Pilot Study

**DOI:** 10.3390/nu14112250

**Published:** 2022-05-27

**Authors:** Wei-Yi Cheng, Wan-Ju Yeh, Jung Ko, Ya-Ling Huang, Hsin-Yi Yang

**Affiliations:** 1 Department of Nutrition, I-Shou University, No. 8 Yida Rd., Jiaosu Village, Yanchao District, Kaohsiung City 82445, Taiwan; wicheng@isu.edu.tw; 2 Graduate Program of Nutrition Science, National Taiwan Normal University, Taipei 116059, Taiwan; wandayeh@ntnu.edu.tw; 3 Department of Applied Biological Chemistry, Graduate School of Agricultural and Life Sciences, The University of Tokyo, 1-1-1, Yayoi, Bunkyo-ku, Tokyo 113-8657, Japan; a532732002@hotmail.com; 4 Department of Laboratory Medicine, E-Da Hospital, No. 1 Yida Rd., Jiaosu Village, Yanchao District, Kaohsiung City 82445, Taiwan; ed105471@edah.org.tw; 5 Department of Nutritional Science, Fu Jen Catholic University, No. 510, Zhongzheng Rd., Xinzhuang Dist., New Taipei City 24205, Taiwan

**Keywords:** dehulled adlay, overweight, obesity, inflammation, lipids

## Abstract

Obesity is a major public health concern worldwide with a rising prevalence. Diets containing whole grains have been demonstrated to benefit body composition and inflammatory conditions in individuals at a high risk of metabolic disorders. This study investigated the effects of dehulled adlay on blood lipids and inflammation in overweight and obese adults. We recruited 21 individuals with abdominal obesity to participate in a 6-week experiment, providing them 60 g of dehulled adlay powder per day as a substitute for their daily staple. Before and after the 6-week intervention, we performed anthropometric analyses and measured blood lipid profiles, adipokines, and markers of inflammation. At the end of the study, the percentage of body fat mass, blood total cholesterol, and triglyceride levels were significantly decreased compared with the baseline. Plasma tumor necrosis factor alpha, interleukin-6, leptin, and malondialdehyde levels were also reduced. In addition, participants with higher basal blood lipid levels exhibited enhanced lipid lowering effects after the dehulled adlay intervention. These results suggest that a dietary pattern containing 60 g of dehulled adlay per day may have a beneficial effect on lipid profiles and inflammatory markers in individuals that are overweight and obese.

## 1. Introduction

Being overweight and obesity are increasing as public health concerns worldwide, with the prevalence of obesity reported to have tripled over the past four decades [1]. Obesity is attributed primarily to the imbalance between energy intake and expenditure, which results in abnormal or excessive fat accumulation [2], and it is considered to be a risk factor for various diseases, including hypertension, coronary heart disease, diabetes mellitus, dyslipidemia, non-alcoholic fatty liver disease (NAFLD), and cancer [3]. The state of obesity is considered to be related to low-grade chronic inflammation and adipocytes that are able to synthesize and release a wide variety of pro-inflammatory cytokines and adipokines during this state and nutrient overload [4]. The production of pro-inflammatory molecules by adipose tissue and continuous inflammation may play a key role in accelerating the progression of metabolic diseases and complications, such as cardiovascular disease and type 2 diabetes mellitus [5]. However, in addition to dietary calorie intake, different dietary patterns may contribute to obesity through their pro-inflammatory effects. The Nurses’ Health Study revealed that a prudent dietary pattern, including a higher intake of whole grains, fruits, vegetables, legumes, fish, and poultry, was inversely associated with plasma C-reactive protein levels compared with Western dietary patterns [6].

Diets containing more whole grains are considered beneficial for individuals with obesity because such diets modulate metabolic disorders and pro-inflammatory states [7]. Adlay (*Coix lachryma-jobi* L. var. *ma-yuen* stapf) is one of the most popular dietary grains worldwide and is also used as a traditional Chinese medicine because of its antioxidative and anti-inflammatory potential [8]. In animal studies, adlay has reduced fat accumulation and modulated lipid metabolism and adipokine excretion [9,10]. A recent study reported that the adlay bran is rich in phenolic compounds that contributes to its anti-inflammatory activities [11]. In RAW 264.7 cells, coixol extracted from adlay reduced proinflammatory cytokine expression through the inhibition of lipopolysaccharide-induced inflammation [12]. Adlay bran-derived polysaccharides have also exhibited a protective effect on Caco-2 cells against tumor necrosis factor alpha (TNFα)-induced epithelial barrier dysfunction [13].

Our previous study revealed that diets containing dehulled adlay ameliorated hepatic steatosis and inflammation in a high-fat-high-fructose diet-induced NAFLD rat model [14]. In addition, a previous study noted that the daily consumption of 60 g of adlay for 4 weeks improved lipid profiles in patients with hyperlipidemia [15], and another recent study demonstrated that ingesting 40–55 g of a formula containing dehulled adlay powder and resistant starch for 12 weeks improved hyperlipidemia [16]. However, the current literature still provides little insight into the physiological effects of dehulled adlay, and clinical trials have been conducted to determine the anti-inflammatory effects of dehulled adlay in adults with obesity. Therefore, this study investigated the effects of dehulled adlay consumption on lipid metabolism and inflammation in adults with overweight and obesity.

## 2. Materials and Methods

### 2.1. Participants

**Figure 1:**
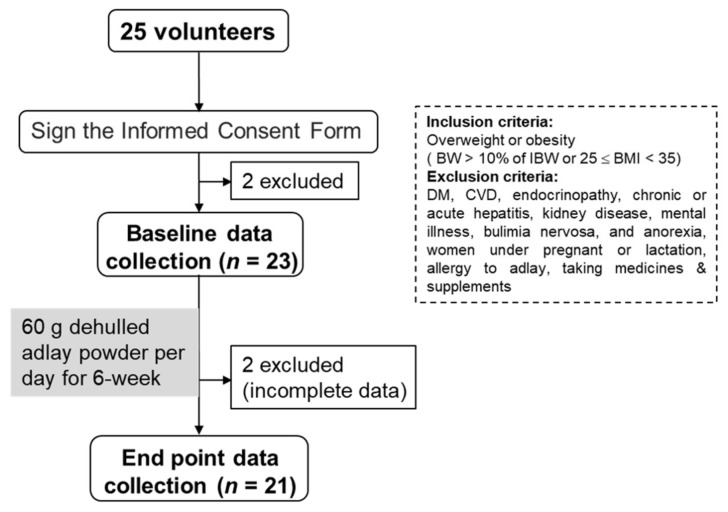
Flow chart of the study protocol. BW, body weight; IBW, ideal body weight; DM, diabetic mellitus; and CVD, cardiovascular disease.

This study was conducted in the College of Medicine, I-Shou University, Taiwan. Twenty-five college students that were overweight or obese (body weight >10% of the ideal body weight (IBW) or 25 ≤ body mass index (BMI) < 35, according to the standards set by the Ministry of Health and Welfare, Taipei, Taiwan) were recruited through printed posters for participation in a 6-week evaluation. All the participants were free of diseases that might interfere with the results, such as diabetes, cardiovascular disease, endocrinopathy, chronic or acute hepatitis, kidney disease, mental illness, bulimia nervosa, anorexia, allergy to adlay, and taking medications or supplements. Female participants who were pregnant or lactating were excluded. This study was approved by the Institutional Review Board of E-Da Hospital (EDAH IRB No.: EMRP-104-138). We explained the purpose, process, and possible risks of the trial to all participants, and they all completed and signed an informed consent form before joining the study. Two of the participants did not meet the inclusion criteria and two did not complete all the sample collections for personal reasons (Figure 1).

### 2.2. Study Design

This was a prospective, single-arm interventional study. A commercial instant dehulled adlay powder (Taichung No. 3, Shi-Field Dreams Farm, Nantou, Taiwan) was used as a dietary whole-grain supplement and each 100 g powder contained 77 g carbohydrate, 1.8 g lipids, and 18.2 g protein. Each participant consumed 60 g of adlay powder per day for 6 weeks as a substitute for their staple as part of any daily meal. At weeks 0 and 6, we performed the anthropometric measurements and collected blood samples for biochemical assessment. All the participants received a dietary consultation with a dietitian at the beginning of the experiment, which they were advised on the use dehulled adlay powder instead of other refined grains in daily meal patterns. The participants were also asked to maintain their dietary habit and physical activity levels. During the experiment, participants returned to the research unit once a week, returning their empty containers to receive the dehulled adlay powder for the following week, and participate in a dietary consultation, which was tailored to individual circumstances.

### 2.3. Dietary Record and Nutrients Analysis

The dietary intake of the participants was evaluated using a 3-day dietary record at weeks 0 and 6. All participants were instructed on how to record the type, amount, and preparation methods of the food they consumed for any 2 weekdays and 1 weekend day. The dietary records were assessed using food scales and models to increase the accuracy of the reported portion sizes, and the data were reviewed and coded by a trained nutritionist. Nutrient compositions were then analyzed using EKitchen software (Nutritionist Edition, Enhancement plus 3, version 2009, Taichung, Taiwan).

### 2.4. Anthropometric Measurements

The anthropometric parameters of all participants, including height, weight, waist circumference, hip circumference, triceps skinfold, and fat-mass, were measured before and after the intervention period. BMI was calculated as weight (kg) divided by height (m^2^). Waist circumference was measured to the nearest 1 cm by using an inelastic measuring tape at the minimum circumference between the lower margin of the last rib and the superior iliac crest. A TANITA TBF 410 (Tanita, Tokyo, Japan) was used to measure body weight to the nearest 100 g as well as to estimate body composition through bioelectrical impedance analysis. Skinfold thickness data were collected with a calibrated Harpenden caliper, accurate up to 0.2 mm. To avoid errors, skinfold measurements were repeated two or three times, with a maximum allowed deviation of 5%.

### 2.5. Blood Sampling and Biochemical Analysis

Blood samples were collected in tubes with or without anticoagulants by medical technologists between 8 and 10 AM after overnight fasting for 8 h at weeks 0 (baseline) and 6 (end). After centrifugation, plasma and serum samples were used to assess biochemical parameters according to the standard procedure of the Department of Laboratory Medicine of E-Da Hospital. The biochemical parameters were fasting glucose, serum C-peptide level, serum triglyceride (TG), total cholesterol (TC), low-density lipoprotein cholesterol (LDL-C), high-density lipoprotein cholesterol (HDL-C), aspartate aminotransferase (AST), and alanine aminotransferase (ALT).

### 2.6. Blood Inflammatory Biomarkers Analysis

The plasma concentration of TNFα, interleukin (IL)-6, IL-10, leptin, adiponectin, angiotensinogen, and irisin was measured using an enzyme-linked immunosorbent assay kit (eBioscience BMS223HS, BMS213HS, BMS215HS, Thermo Fisher Scientific, Waltham, MA, USA; Biovendor RD191001100, Brno, Czech Republic; Assaypro EA3501-1, St Charles, MO, USA; IBL International 27412, Hamburg, Germany; Biovendor RAG018R, Brno, Czech Republic). To determine malondialdehyde (MDA) levels, plasma samples were measured using the thiobarbituric acid-reactive substance (TBARS) method [17]. Briefly, we mixed samples with 20% acetic acid (pH 3.5), 0.8% thiobarbituric acid, and 8.1% sodium dodecyl sulfate and incubated the mixture at 95 °C for 1 h. After the mixture had cooled down to room temperature, trichloroacetic acid was added and detected spectrophotometrically at 532 nm.

### 2.7. Statistical Analysis

All analyses were conducted using SPSS software (version 21.0; IBM SPSS Inc., Chicago, IL, USA). Data are presented as mean ± standard deviation (SD). The differences between the measurement values from weeks 0 and 6 and the differences between the values of the different sexes or subgroups were compared using unpaired *t*-tests. A *p* value of <0.05 was considered statistically significant.

## 3. Results

### 3.1. Diet Composition and Anthropometric Measurements

Twenty-one participants, including 6 men and 15 women, completed the trial, and the average age of the participants was 21.5 ± 1.9 years. The participants returned to the research unit every week with the empty container during the experimental period to get dehulled adlay powder packs for the next week, and they replaced their daily refined grain products with dehulled adlay powder either in drinks or meals in line with the dietitian’s guidance. We detected no difference in daily energy intake and nutrient composition before and after the dehulled adlay intervention and no adverse effects during the experiment were reported. These results indicate that the participants consumed the dehulled adlay powder as a substitute for part of their daily grain food, as advised. In addition, we noted that dietary fiber intake increased significantly from 9.7 ± 7.0 to 19.1 ± 4.8 g/day after the 6-week dehulled adlay intervention. No significant change in body weight was observed at the end of the study, but the body fat mass decreased compared with at baseline (Table 1). In addition, we discovered that the reduction in body weight and fat mass was more evident in women than in men (Table 2).

### 3.2. Biochemical Analysis

**Figure 2:**
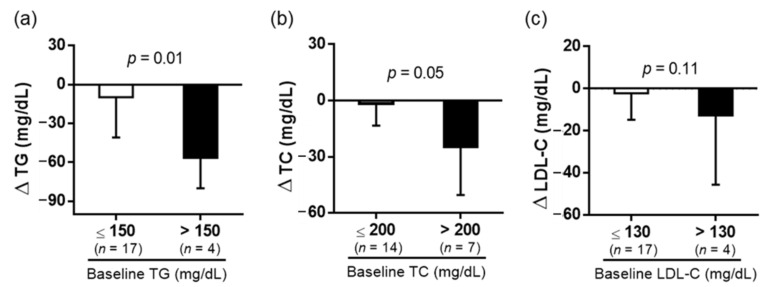
Subgroup analysis according to the baseline TG, TC, and LDL-C levels. Change of (**a**) TG (**b**) TC and (**c**) LDL-C in different subgroups. Values are presented as mean ± SD. Unpaired *t*-test was used to compare the means of baseline and end-of-the study measurements. Significant differences: *p* < 0.05. TC, total cholesterol; TG, triglycerides; and LDL-C, low-density lipoprotein cholesterol.

After 6 weeks of dehulled adlay powder consumption, the serum TC and TG were significantly decreased (Table 1), and no sex difference in relation to changes in serum lipids and hepatic transaminase was revealed (Table 2). Although we noted a slight increase in c-peptide and ALT, the data were all within normal range. We further divided the participants into subgroups according to their blood lipid levels in accordance with the guidelines for TG, TC, and LDL-C levels [18] and discovered that the effects of dehulled adlay consumption on changes in TG and TC levels were more obvious in participants with higher baseline levels (Figure 2).

### 3.3. Blood Inflammatory Biomarkers Analysis

**Figure 3:**
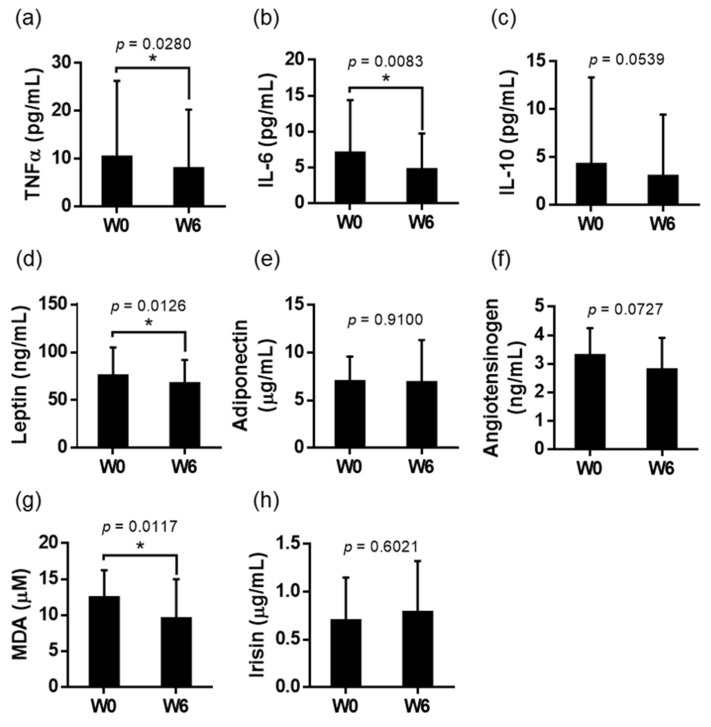
Plasma (**a**) TNFα, (**b**) IL-6, (**c**) IL-10, (**d**) leptin, (**e**) adiponectin, (**f**) angiotensinogen, (**g**) MDA, and (**h**) irisin levels of all participants at baseline (W0) and the end (W6) of the study. Values are presented as mean ± SD. Unpaired *t*-test was used to compare the means of baseline and end-of-the study measurements. * Significant differences compared to W0 (*p* < 0.05). TNFα, tumor necrosis factor alpha; IL, interleukin; and MDA, malondialdehyde.

**Figure 4:**
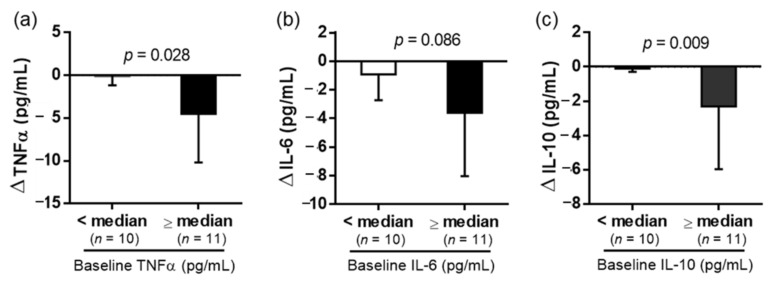
Subgroup analysis according to the baseline (**a**) TNFα, (**b**) IL-6, and (**c**) IL-10 levels (<median, *n* = 10; ≥median, *n* = 11). Values are presented as mean ± SD. Unpaired *t*-test was used to compare the means of baseline and end-of-the study measurements. Significant differences: *p* < 0.05. TNF, tumor necrosis factor; and IL, interleukin.

At the end of the 6-week experimental period, the participants’ plasma TNFα, IL-6, and IL-10 concentrations were significantly decreased compared with at baseline (Figure 3). Individuals with higher baseline TNFα levels had a more obvious effect of reducing the inflammatory status (Figure 4). In the adipokine and TBARS analysis, plasma leptin and MDA levels were also significantly lowered at the end of the experiment, whereas no difference was identified in adiponectin and irisin levels. Although the dehulled adlay consumption tended to lower plasma angiotensin levels, no significant difference was detected (*p* = 0.07) (Figure 3). In addition, no difference was observed between sexes in terms of changes in hsCRP after the 6-week dehulled adlay consumption (Table 2).

## 4. Discussion

Obesity and abnormal body composition are recognized as major factors in metabolic disorders and the progression and mortality of various chronic diseases [19]. Recently established dietary guidelines in Taiwan encourage people to consume more whole grain foods, which are rich in nutrients, dietary fiber, and phytochemicals. The consumption of whole grain foods is related to a lower risk of developing hyperlipidemia, cardiovascular diseases, and diabetes because dietary patterns with a higher intake of whole grain foods have been demonstrated to reduce body fat mass and waist circumference and improve impaired insulin sensitivity and dyslipidemia [20]. One study demonstrated that a higher intake of whole grain than of refined grain led to a lower BMI and waist-to-hip ratio and TG concentrations in adults, which may be partly attributed to the higher dietary fiber content of whole grain food [21]. However, clinical studies using adlay are limited. Previously, Yu and colleagues reported that consuming 60 g of adlay per day for 4 weeks ameliorated hyperlipidemia [15]. In our study, we used a locally grown grain that is easy to obtain and adapt to, and we observed the improvements in blood lipid, adipokine, and proinflammatory parameters after a 6-week consumption of 60 g dehulled adlay powder. In addition, the higher levels of dietary fiber consumption during dehulled adlay intervention and daily nutrient composition remained unchanged after the intervention. A recent study demonstrated that consuming dehulled adlay formula rich in resistant starch and phytochemicals for breakfast for 12 weeks improved the lipid profiles of participants with hyperlipidemia [16]. Our results therefore suggest that increased dehulled adlay consumption as a substitute for part of the daily grain intake can increase dietary fiber intake and have a beneficial effect on body composition and metabolic disorders in adults that are overweight and obese.

A recent study demonstrated a negative correlation between fiber intake and TC and HDL-C ratios [22], and another study indicated that an increased dietary fiber intake from oat bran did not reduce blood TC levels among participants without hypercholesterolemia [23]. Yu et al. reported that receiving 60 g of adlay per day improved plasma lipid profiles in patients with hyperlipidemia [15]. In rats with diet-induced NAFLD, a diet containing dehulled adlay equal to a 60-kg human intake of approximately 60 g per day reduced weight increase, plasma lipids and hepatic proinflammatory cytokines [14]. In our study, we noted that participants ingesting meals containing 60 g of dehulled adlay exhibited reduced blood lipids after the 6-week intervention, especially those with higher basal blood lipid levels. In addition, we observed a decrease in proinflammatory cytokine concentrations. Sex may also be a factor in intervention effectiveness, but studies have reported inconsistent results for sex differences in anthropometric characteristics and inflammatory parameters [24,25,26]. Although some studies have reported that the pathogenesis and therapeutic effects may be related to sexual dimorphism in metabolic diseases such as NAFLD and obesity [27,28], we detected no difference in the metabolic parameters between male and female participants. This may be explained by the small sample size, relatively young age of our participants, and the short duration of the intervention.

The anti-oxidative and anti-inflammatory activities of adlay can be attributed to the flavonoids and various polyphenols in adlay bran [11,29]. In addition, whole-grain foods are dietary sources of nutrients that may potentially affect various metabolic processes [30], and thus, the replacement of daily staple foods with dehulled adlay, containing 3–5 times the vitamin B and E of polished rice according to the Taiwan Food Composition Databases, may have some beneficial effects on regulating metabolic disorders. A study demonstrated that ethanolic extracts in adlay bran inhibited reactive oxygen species (ROS) and pro-inflammatory substance production in vitro [31]. Inflammatory status and oxidative stress are considered to play key roles in the progression of metabolic disorders and complications in obesity [32]. In this study, we revealed that consuming 60 g of dehulled adlay powder per day for 6 weeks significantly decreased lipid peroxidation and circulatory pro-inflammatory cytokine concentrations, especially in those individuals with higher baseline pro-inflammatory cytokine levels. The composition of adlay and its bran exhibits antioxidative and anti-inflammatory activities [29], and a study revealed that the daily intake of adlay as a substitute for polished rice for 4 weeks significantly increased antioxidative capacity in patients with hyperlipidemia [15]. An in vitro study also demonstrated that adlay bran extract reduced the excretion of TNFα and IL-6 in basophilic leukemia cells, which is consistent with our results [31]. Therefore, our results suggest that the dietary consumption of dehulled adlay may help slow the progression of metabolic disorder-related chronic disease by suppressing oxidative stress and inflammatory responses.

Adipokines play a crucial role in regulating the inflammatory response in adipose tissues during the development of obesity and in response to systemic inflammation [33]. Studies have observed an imbalance in adipokines in patients with abdominal fat accumulation. The secretion of adipokines activates inflammatory signaling pathways, eventually leading to the pathogenesis of systemic inflammation and metabolic abnormalities [34,35]. The function of adipose tissue is not only storing energy but also serving as an endocrine organ that secretes bioactive adipokines such as leptin, adiponectin, and TNFα, which play important roles in regulating appetite, metabolism, and inflammation in vivo. Circulatory leptin, primarily secreted by white adipose tissue, is associated with total body fat mass [36]. Plasma leptin levels and total fat mass in our participants were reduced after consuming dehulled adlay for 6 weeks. These results are consistent with those other reports demonstrating that the circulatory leptin levels can be affected by changes in body composition caused by diet modification [37]. Leptin also exhibits pro-inflammatory potential through the stimulation of neutrophil and macrophage activation and increases the secretion of nitric oxide and pro-inflammatory cytokines [38]. By contrast, adiponectin, another adipokine, which is negatively associated with fat mass and abdominal fat, can inhibit the activation of NF-κB, regulate glycemic balance and fatty acid utilization, and protect against inflammation and cardiovascular diseases [39,40]. In addition, white adipose tissue is the main extrahepatic source of angiotensinogen, especially in individuals with obesity [41]. The elevation of circulatory angiotensinogen promotes the formation of angiotensin II, which not only leads to the contraction of blood vessels but also simulates a pro-inflammatory phenotype in adipose tissue, increasing the secretion of TNFα, IL-1β, and IL-6 and decreasing the secretion of IL-10 [42]. Therefore, our results suggest that dehulled adlay may ameliorate the inflammatory status of individuals with obesity by improving the impaired adipokine balance.

This is a single-arm study investigating the effects of dehulled adlay on body composition and lipid metabolism and characterizing its influence on adipokine balance and inflammation in participants that are overweight and obese. This study still has some limitations because of its small sample size, especially after subgrouping, and the relatively short-term duration of the experiment. However, the progression of metabolic diseases is long and gradual, and the study provides preliminary evidence demonstrating that the consumption of dehulled adlay has the potential for preventing the development of metabolic dysfunction-related chronic diseases by improving impaired adipokine balance and decreasing pro-inflammatory cytokine generation in adults who are overweight and obese. In addition, a recent study demonstrated that the effects of adlay intervention may be more pronounced in the older population [16]. In hyperlipidemic and diabetic animal models, adlay derived polyphenols and polysaccharides have also exhibited a protective effect in relation to metabolic disorders through the modulation of gut microbiota [10,43]. A future randomized-controlled study calculating proper sample size based on the pilot study, using a control group consuming refined cereal, and extending the duration of the intervention is suggested, and offering prepared meals for the participants and a more rigorous physical activity assessment may also contribute to eliminate possible confounding factors. Therefore, increasing the number and age range of participants and analyzing gut microbiota may help to increase the study power to achieve more influential results, particularly in relation to the mechanisms underlying making dietary recommendations for the overweight and obese population.

## 5. Conclusions

Our results suggest that a daily intake of 60 g of dehulled adlay has a beneficial effect on blood lipids and the modulation of chronic inflammation in adults who are overweight and obese. Future studies should further clarify the possible underlying mechanisms for the consumption of dehulled adlay as a dietary approach to obesity-related metabolic disorders.

## Figures and Tables

**Table 1:** Anthropometric, blood lipid, and inflammatory parameters for all participants (*n* = 21) at baseline and the end of the study.

Parameters	W0	W6
Height (cm)	162.3 ± 7.4	
Weight (kg)	78.5 ± 9.1	78.6 ± 8.9
Fat mass (%)	40.9 ± 6.9	37.7 ± 6.0 *
BMI	29.8 ± 3.2	29.9 ± 2.9
Waist (cm)	92.0 ± 7.0	90.4 ± 8.4
Hip (cm)	108.9 ± 5.7	108.2 ± 4.7
WHR	0.8 ± 0.1	0.8 ± 0.1
Tricep (mm)	27.7 ± 6.4	25.5 ± 4.2
Fasting glucose (mg/dL)	93.8 ±16.5	91.6 ± 8.2
C-peptide (ng/dL)	2.0 ± 0.5	2.3 ± 0.4 *
HbA1c (%)	5.4 ± 0.9	5.3 ± 0.6
TC (mg/dL)	180.1 ± 34.5	170.8 ± 32.3 *
TG (mg/dL)	114.0 ± 52.3	95.4 ± 43.1 *
HDL-C (mg/dL)	45.8 ± 8.0	46.3 ± 9.0
LDL-C (mg/dL)	105.7 ± 29.6	101.5 ± 30.2
AST (U/L)	20.9 ± 9.0	20.6 ± 12.1
ALT (U/L)	28.7 ± 49.3	32.7 ± 56.8 *
hsCRP (mg/L)	1.88 ± 1.56	2.42 ± 3.38

Values are presented as mean ± SD. Paired *t*-test was used to compare baseline and end-of-the study measurements. * Significant differences: *p* < 0.05. BMI, body mass index; WHR, waist-to-hip ratio; HbA1c, hemoglobin A1c; TC, total cholesterol; TG, triglycerides; HDL-C, high-density lipoprotein cholesterol; LDL-C, low-density lipoprotein cholesterol, AST, aspartate aminotransferase; ALT, alanine aminotransferase; and hsCRP, high-sensitivity C-reactive protein.

**Table 2:** Change in blood lipid and inflammatory parameters after the 6-week experimental period according to sex.

Parameters	Change of Parameters	
	Women (*n* = 15)	Men (*n* = 6)
Body weight (kg)	−0.7 ± 2.0	2.2 ± 3.0 *
Fat mass (%)	−3.0 ± 3.8	−0.3 ± 4.4
Fasting glucose (mg/dL)	−3.6 ± 11.8	1.5 ± 2.4
C-peptide (ng/dL)	0.2 ± 0.5	0.5 ± 0.8
HbA1c (%)	−0.2 ± 0.4	0.0 ± 0.2
TC (mg/dL)	−13.5 ± 21.7	1.3 ± 12.8
TG (mg/dL)	−11.5 ± 37.2	−36.5 ± 21.3
HDL-C (mg/dL)	0.5 ± 5.7	0.5 ± 8.0
LDL-C (mg/dL)	−8.1 ± 18.2	5.7 ± 11.9
AST (U/L)	−1.0 ± 3.6	1.7 ± 6.2
ALT (U/L)	1.6 ± 3.4	10.2 ± 14.2
hsCRP (mg/L)	−0.09 ± 1.46	2.13 ± 5.61

Values are presented as mean ± SD. Unpaired *t*-test was used to compare the means of changes between men and women from baseline to the end of the study. * Significant differences: *p* < 0.05. HbA1c, hemoglobin A1c; TC, total cholesterol; TG, triglycerides; HDL-C, high-density lipoprotein cholesterol; LDL-C, low-density lipoprotein cholesterol, AST, aspartate aminotransferase; ALT, alanine aminotransferase; and hsCRP, high-sensitivity C-reactive protein.
